# AFM investigation of APAC (antiplatelet and anticoagulant heparin proteoglycan)

**DOI:** 10.1007/s00216-021-03765-y

**Published:** 2021-11-13

**Authors:** Maximilian Winzely, Annukka Jouppila, Georg Ramer, Laurin Lux, Bernhard Lendl, Karina Barreiro, Riitta Lassila, Gernot Friedbacher

**Affiliations:** 1 Institute of Chemical Technologies and Analytics, Vienna University of Technology, Getreidemarkt 9/164, 1060 Wien, Austria; 2 Helsinki University Hospital, Clinical Research Institute, Helsinki, Finland; 3 Faculty of Medicine, Research Program in Systems Oncology, Helsinki University, Helsinki, Finland; 4 Institute for Molecular Medicine Finland, Helsinki, Finland; 5 Coagulation Disorders Unit, Department of Hematology , Helsinki University Hospital, University of Helsinki, Helsinki, Finland; 6 Coagulation Disorders Unit, Department of Comprehensive Cancer Center, Helsinki University Hospital, University of Helsinki, Helsinki, Finland; 7 Aplagon OY, Helsinki, Finland

**Keywords:** Antiplatelet, Anticoagulant, APAC, Atomic force microscopy, AFM, Photothermal-induced resonance, AFM-IR

## Abstract

Antiplatelet and anticoagulant drugs are classified antithrombotic agents with the purpose to reduce blood clot formation. For a successful treatment of many known complex cardiovascular diseases driven by platelet and/or coagulation activity, the need of more than one antithrombotic agent is inevitable. However, combining drugs with different mechanisms of action enhances risk of bleeding. Dual anticoagulant and antiplatelet (APAC), a novel semisynthetic antithrombotic molecule, provides both anticoagulant and antiplatelet properties in preclinical studies. APAC is entering clinical studies with this new exciting approach to manage cardiovascular diseases. For a better understanding of the biological function of APAC, comprehensive knowledge of its structure is essential. In this study, atomic force microscopy (AFM) was used to characterize APAC according to its structure and to investigate the molecular interaction of APAC with von Willebrand factor (VWF), since specific binding of APAC to VWF could reduce platelet accumulation at vascular injury sites. By the optimization of drop-casting experiments, we were able to determine the volume of an individual APAC molecule at around 600 nm^3^, and confirm that APAC forms multimers, especially dimers and trimers under the experimental conditions. By studying the drop-casting behavior of APAC and VWF individually, we depictured their interaction by using an indirect approach. Moreover, in vitro and in vivo conducted experiments in pigs supported the AFM results further. Finally, the successful adsorption of APAC to a flat gold surface was confirmed by using photothermal-induced resonance, whereby attenuated total reflection-Fourier transform infrared spectroscopy (ATR-FTIR) served as a reference method.

**Graphical abstract:**

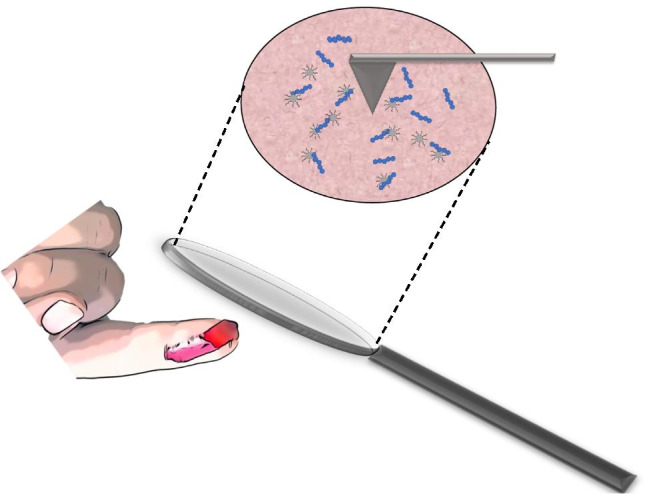

## Introduction

Vascular damage is induced by disease-triggered inflammation or plaque rupture and vessel intervention. The exposed subendothelial matrix components such as collagen, laminin, and fibronectin activate the hemostasis [1]. Platelets respond first by adhering on collagen to seal the injury site. Under arterial shear force conditions, platelet interaction with von Willebrand factor (VWF) is essential for binding and retaining on collagen [2]. VWF is derived from Weibel-Palade bodies of endothelial cells and alpha granules of megakaryocytes or platelets [3–5]. In a resting state, VWF monomers (~ 250 kDa) assemble as coiled bundles up to 20,000 kDa, but at hemostatic challenge, they elongate to long structures with multiple binding sites to endothelial matrix proteins, platelets, and fibrin. VWF also integrates with coagulation system by carrying coagulation factor VIII (FVIII) for protection from degradation and extension of half-life. Further activation of platelets and interplay of coagulation factors finalize a stable fibrin clot. The early development of hemostatic response to injury and the pathological arterial thrombus growth depends on VWF, agonist-induced platelet activation and associated signaling, and a rapid interaction with other extravascular matrix proteins and structures.

The current antithrombotic regime includes systemic antiplatelet agents and anticoagulants for the treatment and prevention of arterial or venous thromboembolism [1, 6]. Antiplatelet agents are directed against platelet activation and aggregation receptors. When treating acute thrombosis, traditional anticoagulant therapy relies on unfractionated heparin (UFH) or low molecular weight heparins (LMWH) which enhance natural anticoagulant of antithrombin to inhibit thrombin and FXa [7].

The beneficial roles in the prevention and treatment of spontaneous and vascular intervention-related thrombosis are well established. To manage arterial thrombosis, both antiplatelets and anticoagulants are needed. Their combined systemic use, however, increases bleeding risk, up to 60–80% with double and 130% with triple therapy. Indeed, targeting the antithrombotic action only to the site where it is needed complies with the requirements of physiological hemostasis.

The adventitial layer of vascular tissue contains mast cells that secrete heparin proteoglycans (Hep-PG) acting as local antithrombotics to control coagulation and vascular repair [8]. Isolated Hep-PG inhibits collagen-induced platelet adhesion and aggregation in the thrombosis models in vitro and in vivo. The novel semisynthetic dual anticoagulant and antiplatelet (APAC) agent (Aplagon Ltd., Helsinki, Finland) was developed to mimic the functional properties of Hep-PG. APAC comprises 5–9-UFH chains (~ 17 kDa) covalently bound to human serum albumin (HSA) core (66 kDa) [9]. APAC inhibits specifically collagen- and thrombin-induced platelet deposition and reduces thrombin and fibrin formation. In several animal models, APAC has been shown to target the damaged vascular sites tightly co-localizing with VWF and laminin, and to reduce acute vessel injury-induced thrombosis [9–12]. APAC is also reno-protective in rat model of acute ischemic kidney injury [13]. While APAC has been studied extensively regarding its functional antiplatelet and anticoagulant activity, further knowledge is still needed on the direct interactions of APAC and hemostatic components, especially VWF.

Motivated by former studies of Bonazza et al. [14], where a successful demonstration of the interaction between VWF and FVIII was achieved, the specific interaction of APAC with VWF was assessed by an indirect approach, using a straightforward drop-casting method to analyze APAC’s size and structure via atomic force microscopy (AFM). In this approach, the species under consideration are mixed in liquid and allowed to react before depositing them on a solid flat surface before AFM imaging. In this way, the ratio between isolated non-reacted APAC molecules and APAC-VWF aggregates can be determined by counting the individual species in the AFM images. With this approach, the adsorption properties of both molecules can be investigated separately and together in more detail with respect to their dimensions on the nanometer and subnanometer scale.

Since identification of species with AFM relies on topographical parameters and comparison with images of blank samples (to exclude misinterpretation by contaminants), a scanning probe–based chemical mid-infrared spectroscopy technique was used to directly verify the chemical identity of the imaged features by chemical spectroscopy on the nanometer scale. The technique, photothermal-induced resonance (PTIR) [15], allows for performing infrared spectroscopy at nanoscale lateral resolution (~ 20 nm) by combining a pulsed, tunable IR-laser with an AFM. The technique acquires mid-IR spectra that are comparable to those seen in bulk FTIR transmission spectroscopy. It not only identifies molecules via their spectroscopic fingerprint, but also has been demonstrated to enable secondary structure analysis of proteins, down to the single-protein level [16–19].

## Materials and methods

### AFM imaging

Samples were drop-casted onto a freshly cleaved mica (muscovite mica V3, Plano GmbH, Wetzlar, Germany). First, a 20-μL droplet of the sample solution was pipetted on the surface and incubated for 5 min. Then, the surface was rinsed for 10 s with Milli-Q® water (18.2 MΩ∙cm). After that, the sample was dried under a constant flow of nitrogen (nitrogen 5.0 from a cylinder) for 30 s.

To prepare APAC sample solutions, the APAC stock solution (containing UFH 7.84 mg·mL^−1^ and HSA 4.08 mg·mL^−1^) was diluted between 1:50 and 1:20,000 with a phosphate-buffered saline (PBS) solution (137 mM NaCl, 2.7 mM KCl, 10 mM phosphate, 7.3–7.5 pH). To prepare VWF sample solutions, 0.6 mg of recombinant VWF (rVWF), vonicog alfa (Takeda Manufacturing Austria AG, Vienna, Austria) was dissolved in 1 mL of PBS. Again, different dilutions were prepared with and without the addition of MgCl_2_ (20 mM).

Tapping mode AFM (TM-AFM) was conducted in air with a Multimode VIII (Bruker, Santa Barbara, CA, USA). N-Doped silicon cantilevers with a spring constant of ~ 40 N/m and a resonance frequency of ~ 300 kHz were used (NCH from Nanosensors, Neuchatel, Switzerland). Imaging was performed at a scan rate of 1 Hz. To verify that surface contaminations are not introduced by the solvents, blank samples containing only buffer and Milli-Q® water were prepared together with the other samples and imaged by TM-AFM as well.

The measured features in the AFM images were evaluated quantitatively with *Gwyddion* [20] with respect to their volume by numerical integration of the AFM data. To quickly and reproducibly select structures, the automatic masking functionality of *Gwyddion* was used to mark aggregates. Cutoffs for heights above the baseline and slopes were chosen to select aggregates and to ignore noise and surface roughness. The *grain distribution* feature enables the user then to easily extract the determined volume for the masked structures.

### PTIR sample preparation and spectroscopy

PTIR imaging requires gold non-IR-absorbing substrates. Here an atomically flat template stripped gold surface (Au.1000.SWTSG; Platypus Technologies, LLC; WI, USA) was used instead of mica. The gold chip was removed from the Si wafer template immediately before sample deposition to minimize surface contamination. Otherwise, sample preparation was carried in the same ways as for AFM imaging.

The sample was measured with a nanoIR3s from Bruker combined with a MIRcat-QT™ mid-IR external cavity quantum cascade laser (EC-QCL) from Daylight Solutions. The measurement was performed in contact mode by using a gold-coated tip (ContGB-G, Budget Sensors) with a radius of curvature of 25 nm and a force constant of 0.2 N/m.

### Infrared spectroscopy (ATR-FTIR)

For comparison of the PTIR results with conventional IR spectroscopy, a reference spectrum of the sample was recorded using the ATR-IR technique with a diamond ATR (Platinum ATR, Bruker) coupled to an FTIR spectrometer (Tensor 37, Bruker). For this measurement, a drop of the APAC stock solution was applied on the internal reflection element (IRE) of the ATR-IR system, and the sample was assessed after evaporation of the solvent.

### In vivo and in vitro porcine arterial injury models and immunostaining

Endovascular balloon injury of porcine iliac artery, and in vitro denudation of femoral artery, followed by tissue collection and immunostaining were as described previously in Barreiro et al. [11]. Briefly, for immunofluorescence staining, the tissue-cryo-sections were incubated with anti-VWF antibody (1:500; A0082; DAKO, Glostrup, Denmark) at + 4 °C overnight. Sections were then washed and incubated 1 h, at room temperature with Streptavidin-eFluor®660 (1:250; 50–4317; eBioscience Inc., Affymetrix Inc., San Diego, CA, USA) and secondary antibody conjugated to alexa568 (1:1000; A11011; Invitrogen Corp., Carlsbad, CA, USA). Sections were subsequently stained with Hoechst 33,342 10 mg/mL (Invitrogen Corp.) and mounted in Mowiol-DABCO mounting media. Samples were imaged with Leica (TCS CARS SP8) or Zeiss (LSM 780) confocal microscopes. Figures included in panels correspond to maximum intensity projection of 10 slice images from confocal z-stacks. To enhance contrast, we used linear adjustment in ImageJ (https://imagej.net/).

## Results and discussion

### Optimization of procedure

**Fig. 1:**
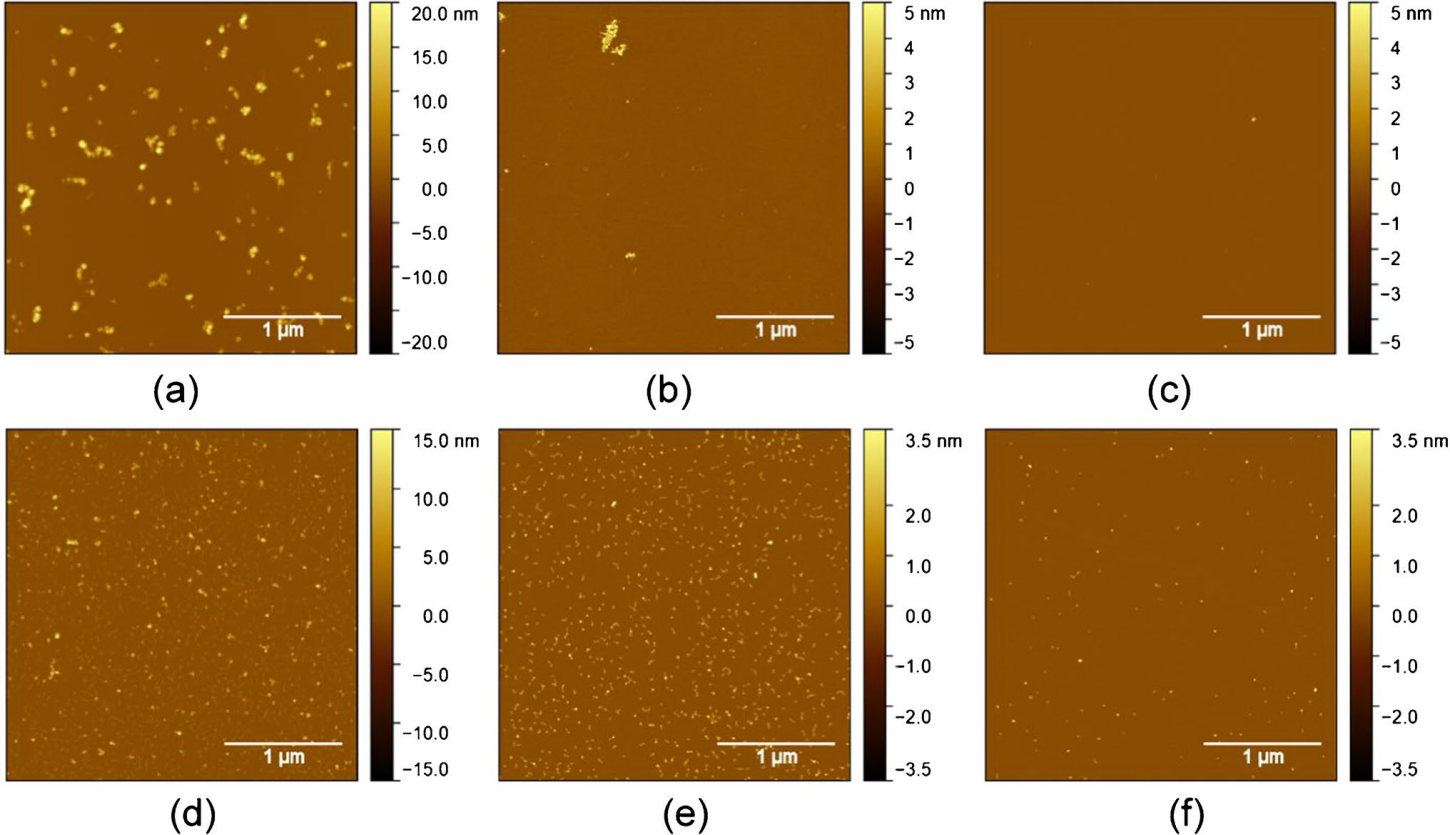
TM-AFM images of APAC drop-casted on mica at various concentrations with and without addition of MgCl_2_. The dilution of the APAC stock solution was **a** 1:50—no MgCl_2_ added; **b** 1:100—no MgCl_2_ added; **c** 1:200—no MgCl_2_ added; **d** 1:100—MgCl_2_ added; **e** 1:1000—MgCl_2_ added; and **f** 1:20,000—MgCl_2_ added

To obtain qualifying AFM images of APAC with the appropriate surface coverage for quantitative evaluation, extensive optimization of the analytical procedure was necessary. For that purpose, drop-casting experiments on freshly cleaved mica with different APAC concentrations, as well as with and without MgCl_2_ as an additive, were performed. Cationic magnesium was chosen, because it is a functionally important basic element for the cellular and molecular interplay of APAC, and other related compounds. The results for different dilutions of APAC with and without MgCl_2_ clearly show that surface coverage was increased with the addition of MgCl_2_ (Fig. 1d–f). Moreover, adsorption of APAC appeared more uniform and less agglomerated than by adsorption from a solution without MgCl_2_ (compare Fig. 1a and d). An explanation for this observation is the surface charge of mica. The mica surface is negatively charged, leading to a repulsion of APAC with its also highly negatively charged sulfonated oligosaccharide groups on the heparin chains. Addition of MgCl_2_ to the adsorption solution leads to a change of the surface charge from negative to positive, thereby enhancing the adsorption of APAC [21, 22].

In fact, we observed that without addition of MgCl_2_, an increase in APAC concentration alone was not sufficient for achieving the desired surface coverage, because in this case, increasing surface coverage was associated with strong agglomeration, which precludes the evaluation of the individual molecules (see Fig. 1a).

Again, the images in Fig. 1 show that sufficiently high surface coverage of APAC without agglomeration can only be obtained under conditions where the negative surface charge of mica is switched to positive by adding 20 mM MgCl_2_ to the adsorption solution.

### Structure-volume

**Fig. 2:**
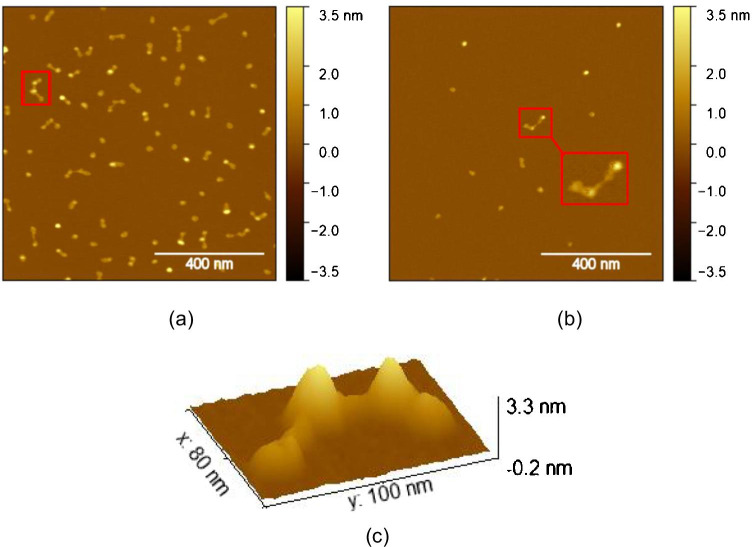
TM-AFM images of APAC drop-casted on mica from a buffer solution containing 20 mM MgCl_2_. The dilution of the APAC stock solution was **a** 1:1000 and **b** 1:20,000. **c** Three-dimensional image of feature marked with the red box in a

To elucidate the structure of APAC in more detail, TM-AFM images with higher magnification were recorded on the samples prepared with a dilution of 1:1000 and 1:20,000 and addition of 20 mM MgCl_2_ (Fig. 2). The APAC molecules observed in TM-AFM images differed in size and shape. Briefly, individual spherical APAC molecules are visible (especially at higher concentration (Fig. 2a) as well as structures that contain multiple APAC molecules connected by chains (Fig. 2c). We interpret this as indication that APAC is not only present in the form of monomers but can also form dimers, trimers, etc. under the used conditions. Furthermore, these chain-connected dots are still visible at lower concentration (Fig. 2b), suggesting their presence in solution and not only as a surface reaction between APAC monomers during the drop-casting.

**Fig. 3:**
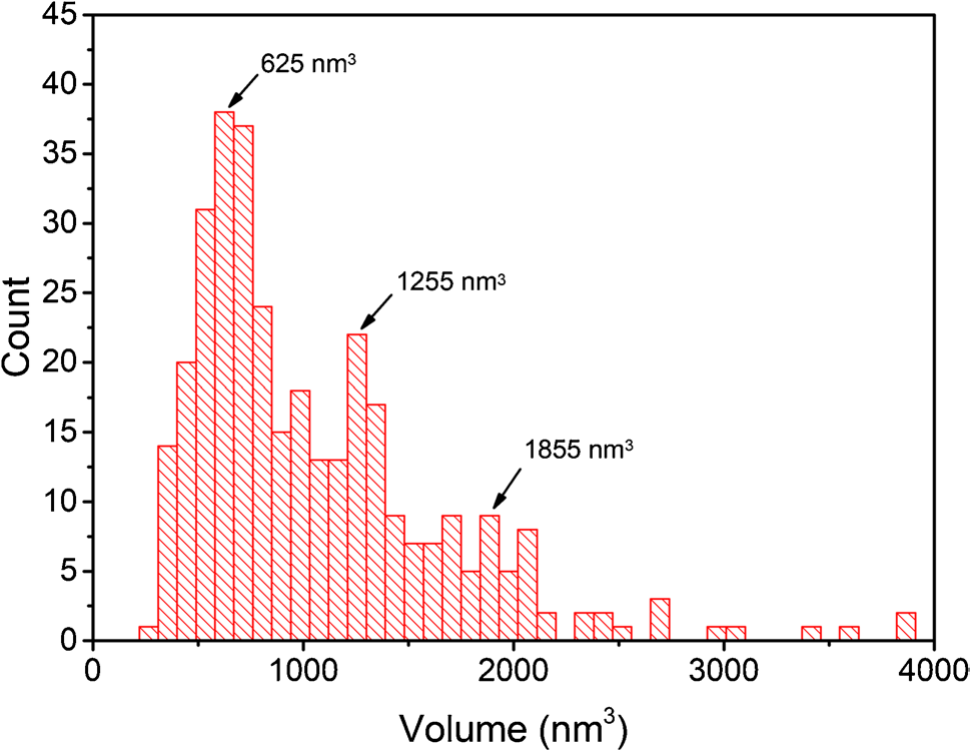
Volume distribution of APAC derived from AFM data

By using the free evaluation software *Gwyddion*, the volume of 340 APAC molecules was determined by selecting the features regarding their height and slope. The result is plotted as a distribution in Fig. 3. By revealing three peaks, the volume distribution further supports that besides monomers, also dimers and trimers are formed, with one subunit exhibiting a volume of about 600 nm^3^ which, assuming a spherical particle, corresponds to a diameter of about 10 nm.

### PTIR spectroscopy

**Fig. 4:**
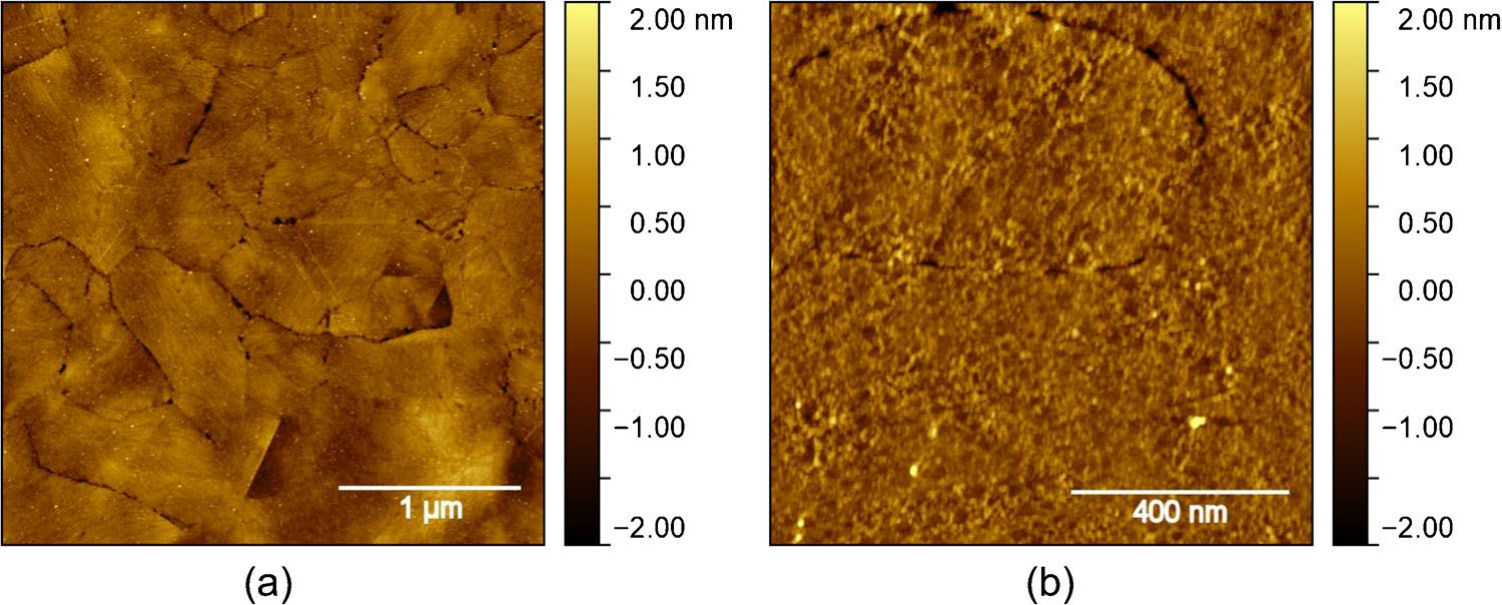
TM-AFM image of a pure gold substrate and b after drop-casting of APAC

PTIR enables to perform mid-IR spectroscopy and imaging at nanoscale spatial resolution. The sensitivity of the technique is sufficient for single-protein spectroscopy [18]. For PTIR measurement, an infrared transparent or reflective substrate is required. As mica absorbs light in the mid-IR range, the sample for the PTIR study was prepared on nearly atomically flat gold instead (see Fig. 4a). A 1:5000 dilution of the APAC stock solution containing 20 mM MgCl_2_ was drop-casted on the fresh gold surface.

Figure 4 shows TM-AFM images of the gold surface before and after drop-casting the APAC solution where the adsorption of individual APAC molecules can be clearly observed by comparison of both pictures.

**Fig. 5:**
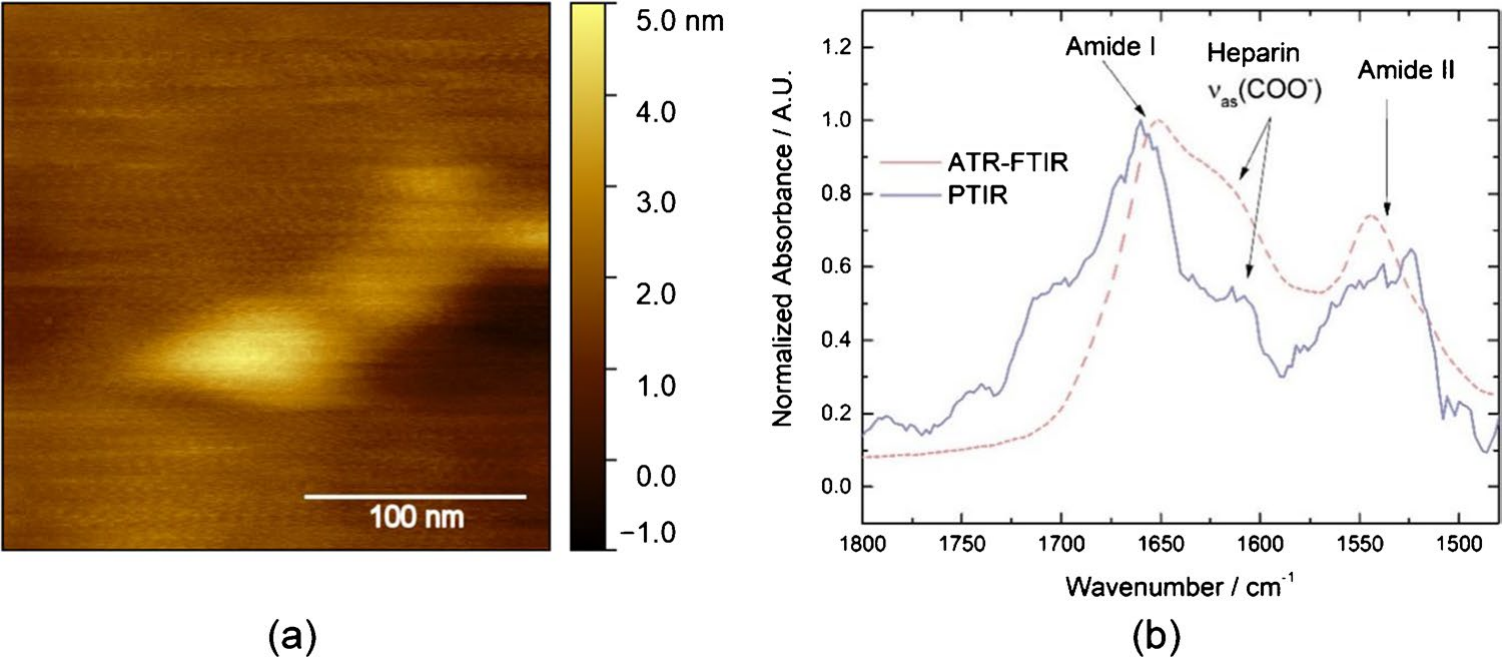
**a** AFM image of APAC adsorbed onto a gold substrate. **b** PTIR spectrum (solid blue line) of the molecule shown in a. ATR-FTIR-spectrum of an APAC reference sample (dashed red line)

Figure 5 depicts the result of PTIR measurements on the gold surface. Figure 5a shows the AFM topography image of an individual APAC molecule recorded on the PTIR-AFM instrument. The apparent larger size of APAC on this instrument compared to TM-AFM can be explained by the higher radius of curvature of the tip used in the PTIR instrument and the higher shear forces exerted on the sample by imaging in contact mode. Figure 5b shows the PTIR spectrum recorded by positioning the tip on the molecule observed in Fig. 5a.

The spectrum reveals the amide I (~ 1660 cm^−1^) and amide II (~ 1540 cm^−1^) bands of the HSA-core. The band positions agree with those found in FTIR spectra of HSA found in literature [23]. Mid-IR spectroscopy of the amide I band also gains insight into the secondary structure of proteins [19, 24]. The position of the band maximum in the range of 1650–1660 cm^−1^ [19, 24] indicates presence of an α-helix structure, confirming the result of a former study about the crystal structure of HSA, exhibiting this protein mainly as an α-helix structure [25]. While the ~ 1630 cm^−1^ band could hint at a presence of β-sheet secondary structure, it may be assigned to the asymmetric carbonyl stretch vibration of UFH [26]. These bands were also detected by ATR-FTIR, where APAC was deposited as a solid on a diamond ATR crystal, confirming the results of PTIR measurements of APAC on gold. The mismatch between spectra can be explained by the difference in sampling volume: the FTIR-ATR spectrum arises from the sum signal of many molecules in the mixture covering the ATR crystal. In this mixture, APAC molecules are present at many slightly different orientations and chemical environments, whereas in PTIR, a single molecule is measured.

### Interaction of APAC with rVWF

**Fig. 6:**
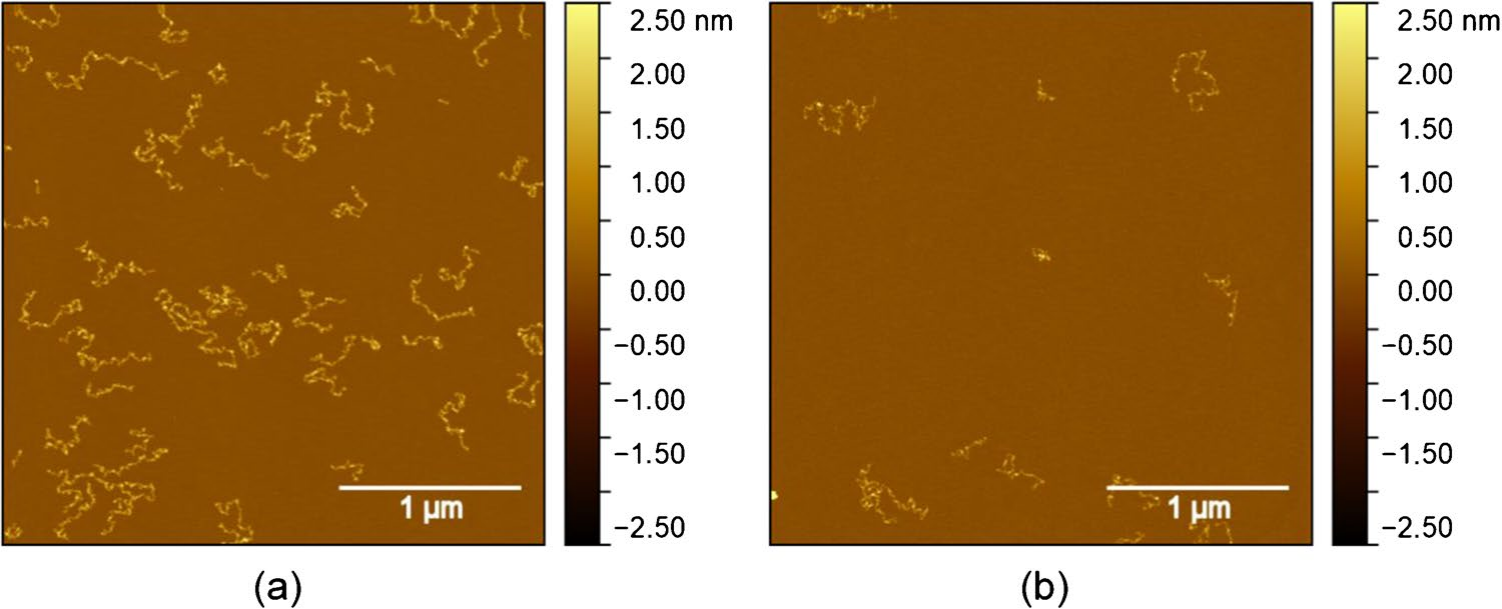
TM-AFM images of rVWF **a** 13.5 μg·mL^−1^ without 20 mM MgCl_2_ and **b** 6.8 μg·mL^−1^ with 20 mM MgCl_2_ drop-casted on freshly cleaved mica

Next, the interaction of APAC with rVWF was investigated with TM-AFM. For that purpose, first, the adsorption behavior of rVWF during drop-casting was investigated and optimized. Figure 6 shows TM-AFM images of rVWF drop-casted on mica both with and without MgCl_2_ in the adsorption solution. Since rVWF possesses a positive charge [27], its properties differ from APAC with respect to adsorption on the negatively charged mica surface. In contrast to APAC, increased adsorption of rVWF is observed on mica with its negative surface charge not reversed by the addition of MgCl_2_ (compare Fig. 6a and b). Nonetheless, adsorption with addition of MgCl_2_ (Fig. 6b), which is needed for the adsorption of APAC [27], is still sufficient for the detection of APAC and rvWF in the mixture. The only difference observed is that the rVWF chains appear to be shorter, when absorbed from solutions containing MgCl_2_ (Fig. 6a).

**Fig. 7:**
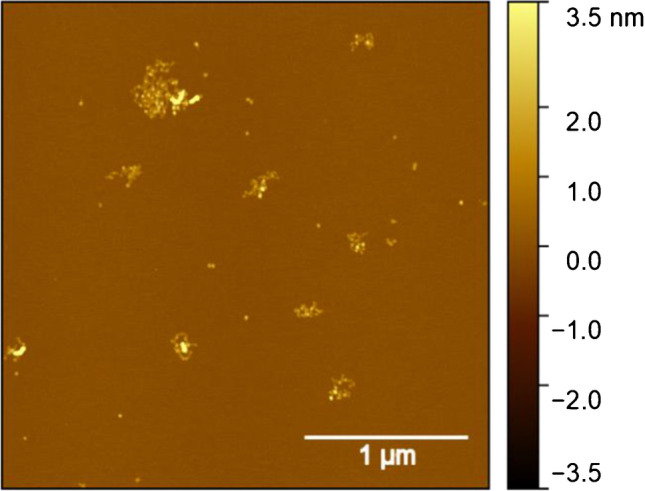
TM-AFM image of a mixture of APAC (1:1000) and rvWF (6.8 μg·mL^−1^) containing 20 mM MgCl_2_ drop-casted on mica

Figure 7 depicts an AFM image of a mixture of APAC (1:1000) and rVWF (6.8 μg mL^−1^) containing 20 mM MgCl_2_ drop-casted on mica. While the rVWF chains are visible, the image is lacking APAC molecules, in contrast to the observation with drop-casting APAC alone (Fig. 1d). This indirect approach suggests that APAC is bound to rVWF, leading to a lower concentration of free APAC in the solution, and thus also a smaller number of individually adsorbed molecules on the mica surface. Furthermore, the absorbed rVWF chains show an increase both in height and in width, further supporting the attachment of APAC molecules.

**Fig. 8:**
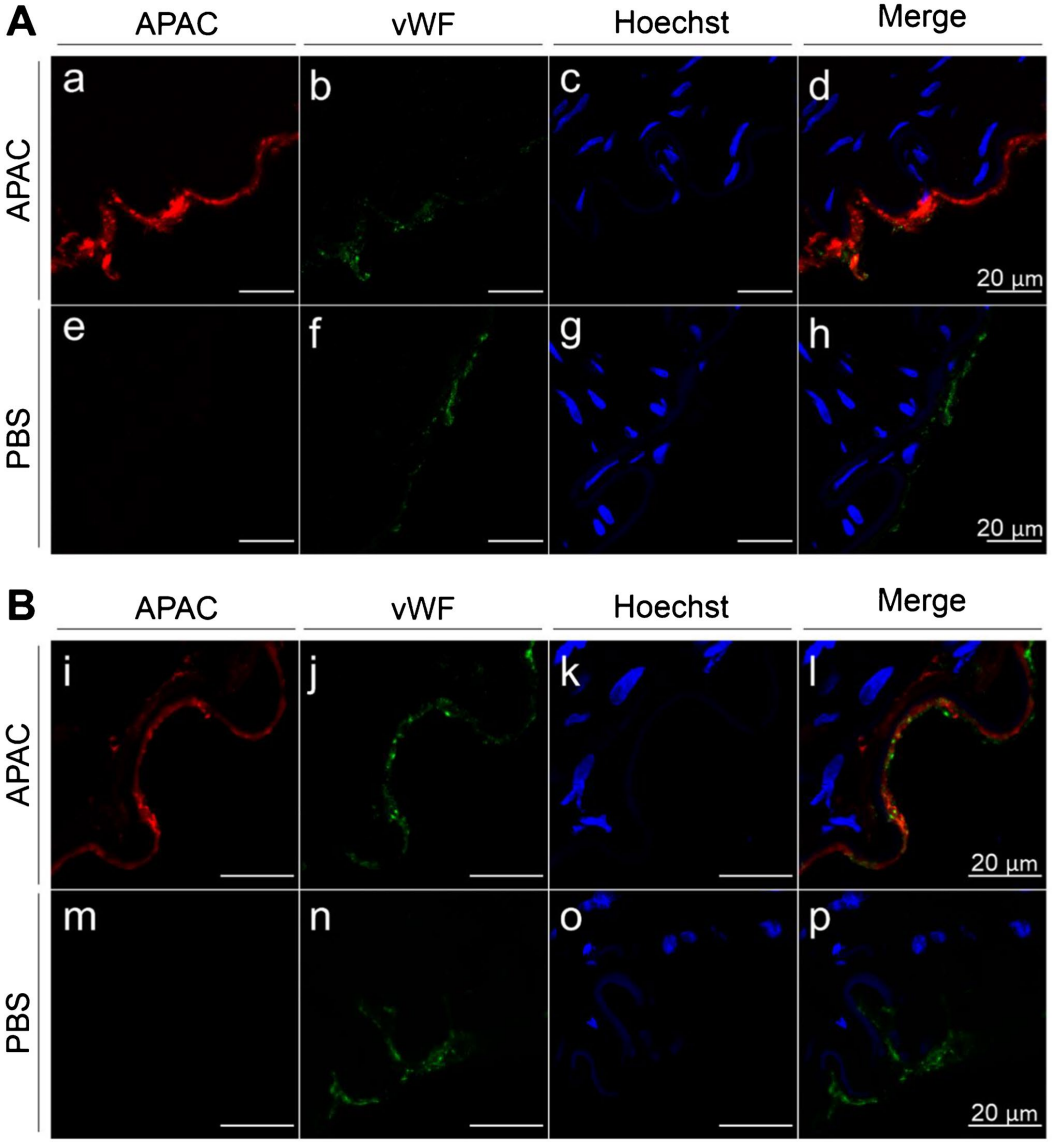
Dual antiplatelet and anticoagulant (APAC) and VWF double staining of arterial luminal sides. Porcine in vitro denuded femoral artery (**A**) and porcine balloon injured iliac artery walls (**B**). In vitro denuded femoral artery and balloon injured iliac artery (before opening the circulation) were incubated with APAC or phosphate-buffered saline (PBS) as control. Histological sections were immuno-stained for APAC (red; a, e, i, m) and for VWF (green; b, f, j, n) and nuclei were stained with Hoechst dye (blue; c, g, k, o). In in vitro denuded femoral artery and balloon injured iliac artery, APAC co-localized with VWF (d, i). In PBS controls, APAC signal was absent (h, p)

These AFM findings are supported by in vitro and in vivo experiments in pigs, where APAC was administered to injured arterial wall (femoral or iliac artery) either in a static contact (in vitro) or under blood flow in a living animal (in vivo) (Fig. 8A and B). In these experiments, APAC was conjugated with biotin to be detected by the fluorescence signal at 650 nm.

APAC was found to target to the elastic lamina and co-localize with VWF (Fig. 8A and B). Previously, in in vivo porcine models of femoral arteriovenous fistula and carotid artery denudation [11], APAC co-localized with VWF. Furthermore, APAC co-localized also with laminin, but not with the intact endothelium [11]. APAC binding to VWF was also proven by the earlier immunoprecipitation studies where recombinant VWF captured APAC from solution [10].

## Conclusion

In summary, AFM was successfully used to characterize the structure and volume of the new promising antithrombotic molecule APAC structure. By optimizing the drop-cast method with respect to concentration and effect of MgCl_2_, the volume of monomeric APAC was determined to be around 600 nm^3^. In addition, under the experimental conditions used, we found that APAC formed oligomers.

During the drug development, several functional platelet and coagulation activation–based assays have been used to identify APAC and its potential as an antithrombotic molecule in the matrix of blood, platelet-rich and platelet-poor plasma, overall, for APAC, a large-sized biological coagulation reaction modifier with naturally occurring heparin proteoglycan as a model. AFM has provided important complementary and visual information on the hemostatically critical cooperation element of von Willebrand factor (VWF), another large-sized biological molecule with multimeric binding sites to APAC.

Furthermore, we were able to chemically identify individual APAC molecules adsorbed to a flat gold surface by means of PTIR, where we used ATR-FTIR equipped with a diamond as internal reflective element for comparison.

Last, but not least, our studies about the interaction between APAC and VWF allowed us to contribute new insight of APAC’s anticoagulant and antiplatelet activities. Thereby, we are providing evidence of the specific binding of APAC to VWF. This result aligns with the in vivo observations of APAC’s ability to decrease platelet recruitment and interaction with the vessel wall.

From the drug development aspect, the described AFM technique will provide insight also into other basic molecular interactions by providing structure–function relationships. The relevant conditions for these interactions can be revealed and modified to unravel the broad binding characteristics of biochemical entities.
